# On Hydrocele of the Neck—A Clinical Note

**Published:** 1875-07

**Authors:** Sampson Gamgee

**Affiliations:** Surgeon to the Queen’s Hospital, Birmingham, England


### ON HYDROCELE OF THE NECK—A CLINICAL NOTE.

BY SAMPSON GAMGEE, F. R. S. E.,

*Surgeon to the Queen's Hospital, Birmingham, England.*

In March last year, Mrs. D——, from Wolverhampton, called on me with her youngest child, a healthy looking boy two years old, who had a tumour on the left side of the neck. The growth was noticed very soon after birth, and had steadily increased to its present size. When the clothes were removed, I found a round smooth mass occupying the whole left side of the neck, and projecting over the clavicle on the upper part of the pectoral region.—Fluctuation and translucency being very distinct, I introduced a trocar at the most dependent part in front, and drew off nearly a pint of pale, straw-colored, and richly albuminous liquid. After closing the aperture with styptic colloid, and applying a cotton-wool compress, I requested to be informed of the progress of the case. I heard nothing of it for eight months. When the child was again brought to me last December, the tumor was larger than when first seen, and the contents, though still liquid, had undergone a bloody change. The mass was no longer translucent, and the skin was uniformly bluish. I introduced two ordinary-sized drainage tubes from back to front, at a distance of a couple of inches, and applied a tenax compress. A considerable quantity of reddish fluid oozed through the tubes, but as days elapsed the mass did not perceptibly lessen, and it became evident that something more must be done to effect a radical cure. Dissection has proved that these congenital cystic growths in the neck are under the fascia; and in the particular case entire removal would only have been possible after a dissection attended with risk. With a view to effect a cure with the utmost safety, I removed the two small drainage-tubes, and while my friend and colleague, Dr. Mackey, administered chloroform, I made an incision on the anterior aspect, a little below the middle line of the tumor, and pushed into its centre an India rubber drainage-tube, two inches long and a quarter of an inch in diameter; the anterior extremity of the tube projected sligtly from the wound, and was kept in position by a loop of thread on each side secured by adhesive plaster. At the end of a week a great deal of irritation had been set up; the mass was hot and semi-solid; the child was feverish, and the discharge semi-purulent. The tube was now removed, and a linseed poultice applied. Within a week three separate collections of matter were evacuated by the aid of the lancet; fever subsided, a dry pad was applied with daily increasing pressure and the rapid decrease of the enlargement. No trace of it is now perceptible, and the child is perfectly well.—*London Lancet.*

---

*Cramp of Telegraph Operators.*—M. Ominus contributes an observation on this affection to the *Gaz. Med. de Paris,* April 10th. After alluding to the cramp due to repeated movements of certain muscles, and observed not only among writers, but also among designers, engravers and musicians, he gives a case of a similar character occurring in a telegraph operator. The patient, who had used the Morse instrument for nineteen years, first noticed a difficulty in making the dots, and especially a succession of dots. The first letters which he found difficulty in forming were *l,* which is indicated by three dots, *i,* which is indicated by two dots, and *u,* which is indicated by two dots and a line. *D,* which is formed by a line and two points, he found easier, because the first movement finishing the line gave greater assurance of movement. Soon it became impossible to make dots at all by the ordinary manipulation, and the patient then had recourse to his thumb, which he used for two years. At the end of that time his thumb was seized with cramps, and he then had recourse to the index and medius successively. Each of these was used two or three months, and then became useless on account of cramp. Even the use of the wrist, which he finally resorted to, gave rise to a trembling in the fore-arm, and at last to the same symptom in the leg of that side, as well as pain in the neck, and sometimes vertigo and insomnia. The only method of relieving these symptoms is to change the instrument employed, using that known as Hughes', alternating this with Morse's.—*Medical Times.*

